# Retraction Note to: Osteoblastic differentiation of stem cells induced by graphene oxide-hydroxyapatite-alginate hydrogel composites and construction of tissue-engineered bone

**DOI:** 10.1007/s10856-021-06549-z

**Published:** 2021-06-11

**Authors:** Xuanze Li, Jiao Chen, Zhe Xu, Qiang Zou, Long Yang, Minxian Ma, Liping Shu, Zhixu He, Chuan Ye

**Affiliations:** 1 Department of Orthopaedics, The Affiliated Hospital of Guizhou Medical University, 550004 Guiyang, China; 2 Key Laboratory of Adult Stem Cell Transformation Research, Chinese Academy of Medical Sciences, 550004 Guiyang, China; 3 National-Local Joint Engineering Laboratory of Cell Engineering and Biomedicine, 550004 Guiyang, China; 4 Center for Tissue Engineering and Stem Cell Research, Guizhou Medical University, 550004 Guiyang, China; 5 China Orthopaedic Regenerative Medicine Group (CORMed), 310000 Hangzhou, China

Retraction to: **Journal of Materials Science: Materials in Medicine (2020) 31:125**

10.1007/s10856-020-06467-6

This article has been retracted at the request of the authors due to the multiple errors in Fig. 13. As some in vivo data are flawed, the conclusions of the article are no longer viable. The authors sincerely apologize to readers and the journal for any inconvenience caused. The authors are repeating their study and will submit a corrected manuscript for peer review. All authors agree to this retraction.

